# Autologous whole-blood or autologous serum acupoint injection therapy for chronic urticaria: A systematic review protocol

**DOI:** 10.1097/MD.0000000000016127

**Published:** 2019-06-21

**Authors:** Leixiao Zhang, Xianjun Xiao, Ruting Hui, Yunzhou Shi, Yanli Deng, Hui Zheng, Qianhua Zheng, Siyuan Zhou, Junpeng Yao, Wei Cao, Ying Liu, Pingsheng Hao, Ying Li

**Affiliations:** a Acupuncture and Tuina School, Chengdu University of Traditional Chinese Medicine, Chengdu; b The People's Hospital of Jianyang City, Jianyang; c Chengdu First People's Hospital; d Sichuan Second Chinese Medicine Hospital; e Clinical Medical College of Chengdu University of Traditional Chinese Medicine, Chengdu, Sichuan, China.

**Keywords:** acupoint, autologous serum, autologous whole-blood, chronic urticaria, injection, protocol, systematic review

## Abstract

**Background:**

Chronic urticaria (CU) is a common and easily recurring skin disease in the world. Many trials have shown that autologous whole-blood or autologous serum acupoint injection therapy is effective in treating CU. There is currently no systematic review of this therapy. The program aims to evaluate the effectiveness and safety of this therapy in patients with CU.

**Methods:**

Literature search will be conducted at Medline, PubMed, Excerpt Medica Database, Springer, Web of Science, Cochrane Library, China National Knowledge Infrastructure, Chinese Scientific Journal Database, and other databases. The search date is until May 2019. We will search for popular terms including CU and this therapy. Import the literature electronically. Duplicate documents will be deleted. The primary outcome is the urticaria activity score or other validated scales. Secondary outcomes included response rate, quality of life scale, recurrence rate, and adverse events. A systematic review and search for a randomized controlled trial of this therapy for CU. Implement the Cochrane RevMan V5.3 bias assessment tool to assess bias assessment risk, data integration risk, meta-analysis risk, and subgroup analysis risk (if conditions are met). The mean difference, standard MD, and binary data will be used to represent continuous results.

**Results:**

This study will provide a comprehensive review of the available evidence for the treatment of CU with this therapy.

**Conclusion:**

This study will provide new evidence for assessing the effectiveness and side effects of this therapy for CU. Since the data is not individualized, there is no need for formal ethical approval.

**PROSPERO registration number:**

CRD42019128364.

## 1 Introduction

### 1.1 Description of the condition

Urticaria, often referred to as “hives,” has a long and rich history, dating back at least to the 10th century BC. When it is called “Feng Yin Zheng” in China.^[1]^ Chronic urticaria (CU) is a common skin disease characterized by a short-term (<24 hours) spontaneous skin rash (urticaria) with or without angioedema that lasts longer than 6 weeks.^[1–5]^ The internationally recognized classification of CU is chronic spontaneous urticaria and inducible urticaria (IU).^[3]^ IU can be divided into physical urticaria according to the complex etiology (including symptomatic sports urticaria, cold urticaria, pressure urticaria, solar urticaria, hot urticaria, vibrating angioedema), cholinergic urticaria, contact urticaria, or waterborne urticaria.^[3]^

A survey found that more than half of the respondents had urticaria symptoms lasting 6 to 10 weeks, and 12% of patients had an outbreak for 52 weeks per year.^[4,6]^ CU is not life-threatening but has been shown to have a significant impact on the physical and mental health of patients. Its impact on the quality of life is comparable to that of severe coronary artery disease and even exceeds that associated with respiratory allergies.^[7]^ Because of chronic itching or physical discomfort in patients with CU, symptoms such as anxiety, insomnia, and psychological stress are prone to occur.^[8]^

Due to the lack of long-term effective treatment, severe intractable symptoms increase the patient's medical expenses.^[9]^ Patients spend an average of $2047 per year, with drugs alone accounting for 62.5% ($1280) of the total annual cost.^[10]^ They spend about $569 a year on evaluation, management, and testing.^[11]^ Urticaria is more common than before and may be increasing. As many as 1 in 4 people have had an urticaria experience in their lifetime. At any time, 0.5% to 1.0% of people have CU. Women between the ages of 20 and 40 (or older) are at higher risk of developing urticaria, which is twice as common as men. The most common form is CU.^[11–13]^ According to a report, the average absenteeism and attendance rates for CU patients were 6.4% and 20.8%, respectively, and the average work efficiency loss was estimated at 20.7%.^[14]^ This means that the prime time of the patient's work is affected.^[4,15]^ In the United States, mental/physical health and work/non-work activities are impaired in CU patients, similar to patients with moderate to severe psoriasis.^[16]^

CU is characterized by persistent wheals or hives and frequently originates from degranulation of cutaneous mast cells leading to extravasation of plasma in the dermis layer.^[17]^ Urticarias are pruritic, edematous erythematous lesions of variable size that blanch under pressure.^[18]^ The primary effector cells in patients with urticaria are mast cells, which are present in high numbers throughout the body, including the subcutaneous tissue. Activated mast cells produce a wide variety of proinflammatory and vasodilatory substances, including the immediate (<10 minutes) release of histamine from granules and the production of leukotriene C4 and prostaglandin D2 from membrane phospholipids. There is also more delayed (4–8 hours) production and secretion of inflammatory cytokines, such as TNF-a, IL-4, and IL-5. The immediate products are responsible for pruritus, swelling, and erythema, whereas the later products lead to an influx of inflammatory cells.^[17–19]^

According to revisions to the international guidelines on the diagnosis and therapy of CU: the most important diagnostic step of CU includes a thorough history, physical examination, and a ruling out of severe systemic disease. A thorough history should include the most possible inducible factors or critical aspects of the nature of the patient's urticaria.^[3]^ Reducing clinical symptoms is the main job of treating CU. First-line treatment options for CU include antihistamines, anti-inflammatory drugs, immunosuppressive agents, monoclonal antibodies against immunoglobulin E, antibiotics, and antidepressants.^[7,20,21]^ The goal is to eliminate potential causes and triggers. Second-line treatment includes symptomatic treatment and other medications.^[3,22]^ In fact, the treatment of CU is recommended in 3 steps. Starting with a standard dose of H1 nonsedating antihistamine, if the treatment response is inadequate, the dose should be increased by a factor of 4. In the treatment of refractory patients, it is recommended to use omalizumab, cyclosporine A or montelukast in the third step. Short-term corticosteroid treatment can be considered for up to 10 days.^[22–24]^ However, the latest study found that patients with CU had a median tolerance to H1-antihistamines of 3 years, inadequate treatment, and impaired quality of life.^[25]^ Long-term use of H1-antihistamines can cause adverse reactions such as headache, lethargy, fatigue, dry mouth, allergies, and so on, especially the first generation of H1-antihistamines (such as diphenhydramine, chlorpheniramine), even at the usual therapeutic doses, there is a sedative effect from mild sleepiness to deep sleep.^[24,26,27]^

### 1.2 Description of the intervention

Autologous whole-blood (AWB) or autologous serum (AS) acupoint injection therapy is a method of repeatedly injecting the patient's own venous whole-blood or serum into the patient's own muscle tissue or acupoints to treat the disease. According to China's ancient medical theory, stimulating acupuncture points is an effective treatment and is used to prevent disease and improve health.^[28,29]^ For example, more and more evidence indicates that effective stimulation of Zusanli (ST36) has immunomodulatory and anti-inflammatory effects,^[29]^ which is one of the important acupoints for regulating the neuro-immune-endocrine network.^[30]^

AWB or AS acupoint injection therapy has been proposed as a treatment for a variety of diseases, such as allergies, inflammation, infections, and autoimmune diseases. The main mechanism is the proinflammatory expression of the patient in the circulatory system, signal tolerance, and desensitization. This is useful in developing countries because it is a cheaper option.^[31,32]^ Acupuncture plus acupoint-injection is effective in treating intractable urticaria, which is more obvious than intramuscular injection in the buttocks.^[33,34]^ Therefore, AWB or AS acupoint injection therapy may be an option for treating CU patients,^[35]^ including pregnancy or lactation.^[36]^

### 1.3 How the intervention might work?

AWB or AS acupoint injection therapy is an ancient treatment method,^[37]^ namely repeated intramuscular injection of AWB or AS. It is can cause double stimulation to the body and acupoints, and regulating the balance of Qi circulation.^[38]^ Our hypothesis was that this therapy reduces the levels and/or effects of these mast cell-degranulating IgE and IgG autoantibodies.^[39,40]^ The mechanism of action of this therapy in patients with CU is largely unknown.^[41]^ Some people think that this therapy can regulate the immune response to autoantigens and desensitization,^[35,42]^ from a Th2 to a Th1 pattern,^[43]^ and also include a reduction in IgE-anti-IL24 autoantibodies, and so on.^[39]^

### 1.4 Why it is important to conduct this review?

According to traditional Chinese medicine (TCM) theory, acupoint stimulation is a complex, ritualistic somatosensory intervention with multiple components.^[44]^ By stimulating acupoint, meridian, and collaterals could be activated to tonify and promote Qi.^[44]^ AWB or AS acupoint injection therapy is an important method of acupoint stimulation. In some countries, such as China, Germany, India, Iran, South Korea, Turkey, Mexico, and the USA, autohemotherapy is also commonly used to treat antihistamine resistant patients with CU.^[39,42]^ It is considered a safe intervention for the treatment of CU, with rapid and sustained beneficial clinical effects. There are some conflicting results about its effectiveness, but it has no serious side effects.^[37,39,41,43]^ Although there is currently no efficacy evaluation and standardized management programs, most Chinese medicine hospitals conduct AWB or AS acupoint injection therapy for CU based on their own experience. It is particularly important to assess its safety and effectiveness, and it is necessary to present treatment recommendations based on available evidence.

### 1.5 Objectives

To develop treatment recommendations, we systematically evaluated the efficacy and safety of AWB or AS acupoint injection therapy for CU.

## 2 Methods

### 2.1 Study registration

PROSPERO registration number is CRD42019128364. This protocol report is structured according to the preferred reporting items for systematic reviews and meta-analyses protocols (PRISMA-P) statement guidelines.^[45]^

### 2.2 Inclusion criteria for study selection

#### 2.2.1 Types of study

In order to evaluate the efficacy of AWB or AS acupoint injection therapy in the treatment of CU, this paper only reviewed the randomized controlled trial (RCT) between AWB or AS acupoint injection therapy and the control group, including drug, no treatment, placebo, diet, and exercise therapy, and so on. In addition, both Chinese and English publications are subject to language restrictions. All RCT that are not subject to publication state constraints will be included. If the experiment shows that the phrase is random and the blind method is not restricted, it will be regarded as a random study. Animal mechanism studies, case reports, self-controlled, non-RCTs, random crossover studies, and quasi-randomized trials will be excluded.

#### 2.2.2 Types of participants

Regardless of gender, age, ethnicity, education, and economic status, patients with CU who meet the following diagnostic criteria (eg, EAACI/GA2LEN/EDF/WAO guidelines, guidelines for the diagnosis and treatment of CU).^[3,46]^

#### 2.2.3 Types of intervention

AWB or AS acupoint injection therapy instruments are based on sterile syringes. It includes either a single AWB or AS acupoint injection therapy or a combination of AWB or AS acupoint injection therapy and antihistamines. It does not include the combination of AWB or AS acupoint injection therapy with different types of TCM adjuvant therapy (such as acupuncture and moxibustion, TCM decoction, etc). A comparison of the following processing will be performed:

(1) Single AWB or AS acupoint injection therapy or a combination of AWB or AS acupoint injection therapy and antihistamines compared with no treatment.
(2) Single AWB or AS acupoint injection therapy or a combination of AWB or AS acupoint injection therapy and antihistamines compared with placebo or sham acupuncture.
(3) Single AWB or AS acupoint injection therapy or a combination of AWB or AS acupoint injection therapy and antihistamines compared with other active therapies.
(4) Single AWB or AS acupoint injection therapy or a combination of AWB or AS acupoint injection therapy and antihistamines compared with the same active therapy.

#### 2.2.4 Types of outcome measures

The primary outcome included urticaria activity score, urticaria control test, or other validated scales used to improve itching and skin symptoms after at least 2 weeks of treatment.^[3,47]^ Secondary outcomes included response rate, quality of life scale (eg, CU-Q2oL, DLQI, etc), recurrence rate during the follow-up period, and adverse events.^[48]^ The system review will be performed independently.

### 2.3 Data sources

Our systematic review will search all RCT for AWB or AS acupoint injection therapy of CU, electronically and manually, regardless of publication status and language, until May 2019. Databases include: Medline, PubMed, Excerpt Medica Database, Springer, Web of Science, Cochrane Library, WHO International Clinical Trials Registry Platform, Traditional Chinese Medicine databases, China National Knowledge Infrastructure, China Biomedical Literature Database, Chinese Scientific Journal Database, and Wan-Fang database. Other sources, including reference lists of identified publications and meeting minutes, will also be searched. Manually search for grey literature, including unpublished conference articles.

### 2.4 Search strategy

The following search terms will be used: randomized controlled trial (RCT); autologous blood therapy (eg, “autologous whole blood” or “autologous serum”); antihistamines (eg, “loratadine” or “cetirizine”); acupoints (eg, “meridian acupoints” or “Gu-Kong” or “Qi-Fu”); urticaria (eg, “chronic urticaria” or “hives” or “nettle-rash” or “Fong-Tzen-Kwai” or “wind-rash-patch” or “Feng Yin Zheng”). The same search term will also be used in the Chinese database. Search strategies based on the Cochrane Handbook guidelines will be implemented in all electronic databases.^[49]^ Medline's search strategy is shown in Table 1.

**Table 1:**
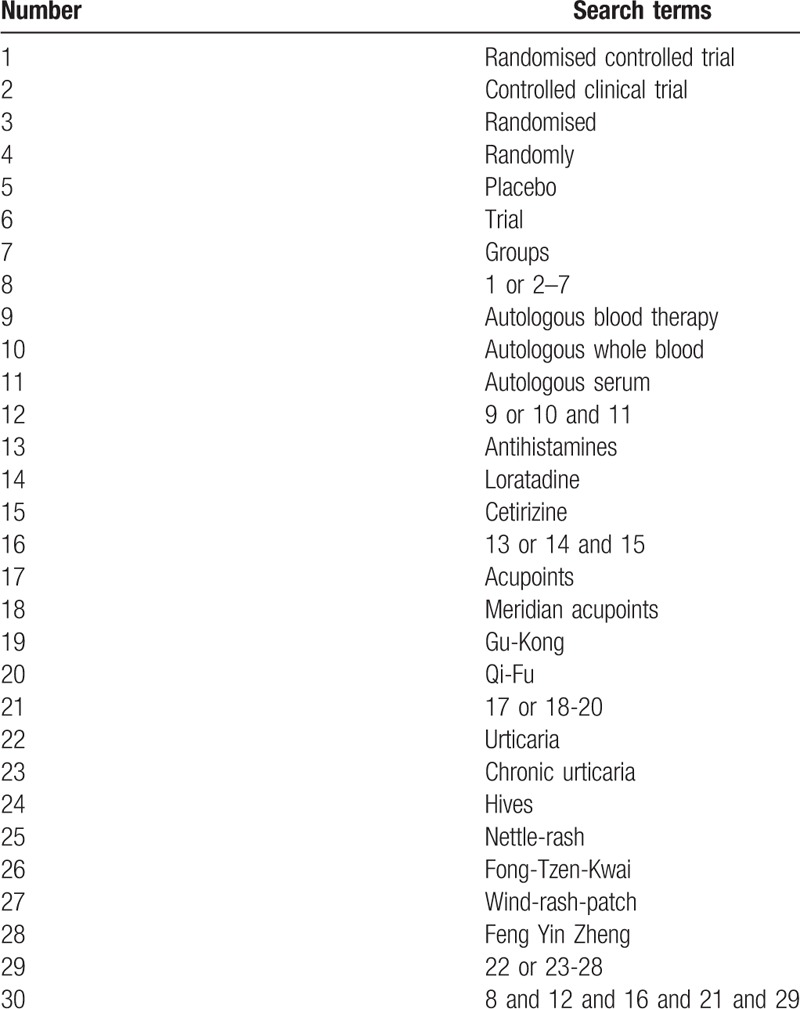
Medline's search strategy.

### 2.5 Data collection and analysis

#### 2.5.1 Selection of studies

**Figure 1:**
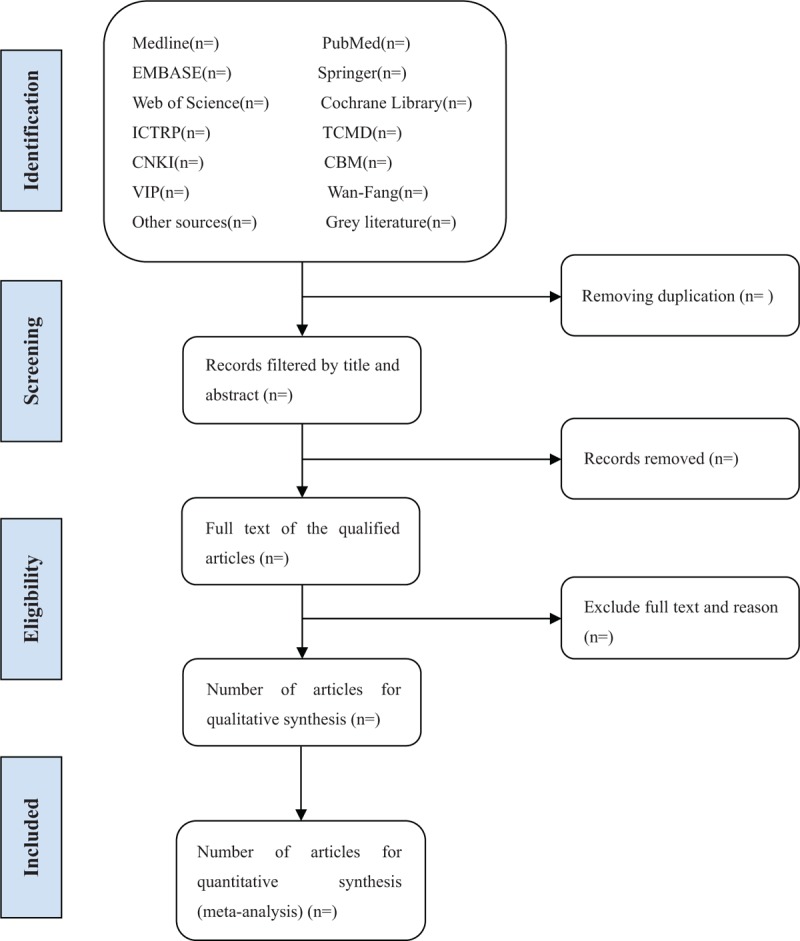
Flow diagram of studies identified.

Before literature retrieval, all reviewers are trained to ensure a basic understanding of the background and purpose of the review. In the literature screening process, we will use EndNote software (V.X8) document management software. The 2 comment author (LXZ and YZS) will be in strict accordance with the inclusion criteria, independent screen all retrieval research, read the title, abstract and keywords in the literature, and determine which meet the inclusion criteria. We will obtain the full text of all relevant studies for further evaluation. Excluded studies will be recorded and explained. If there is a disagreement in the selection process, it will be discussed by 2 authors (LXZ and YZS) and the third author (YL) will arbitrate. If necessary, we will contact the trial author for clarification. The primary selection process is shown in a PRISMA flow chart (Fig. 1).

#### 2.5.2 Data extraction and management

The authors will extract data independently from the selected report or study and fill out the data extraction form. We will obtain data on general information, participants, methods, interventions, outcomes, results, adverse events, conflicts of interest, ethical recognition, and other information. When the reported data is insufficient, we will contact the author for further information. Any differences will be resolved through discussions between the 2 authors, and further differences will be arbitrated by the third author (YL).

#### 2.5.3 Assessment of risk of bias and reporting of study quality

The authors (XJX and RTH) will use the Cochrane Collaboration's bias risk assessment tool to assess the risk of bias in all included studies. We will assess the risk of bias in the following areas: sequence generation, assignment sequence hiding, the blindness of participants and staff, and result evaluators, incomplete outcome data, selective outcome reporting, and other sources of bias. This review uses L, U, and H as the key to these assessments, where L (low) indicates a lower risk of bias, U (unclear) indicates that the risk of bias is uncertain, and H (high) indicates a higher risk of bias. If inconsistent results appear, the final decisions will be made by the third author (YL). Information on the risk of biased assessments included in the study is summarized in tabular form and the results and impacts are critically discussed. If the information is ambiguous, we will try to contact the author. For repeated publications, we only select the original text.

#### 2.5.4 Measures of treatment effect

Data analysis and quantitative data synthesis will be performed using RevMan V.5.3. For continuous data, if there is no heterogeneity, we will use mean difference (MD) or standard MD to measure the therapeutic effect of 95% confidence interval (CI). If significant heterogeneity is found, a random effects model will be used. For dichotomous data, we will use the 95% CI risk ratio for analysis.

#### 2.5.5 Unit of analysis issues

We will include data from parallel group design studies for meta-analysis. Only the first phase of the data will be included in the random crossover trial. In these trials, participants were randomly divided into 2 intervention groups and individual measurements for each outcome of each participant were collected and analyzed.

#### 2.5.6 Management of missing data

If the primary result has missing or incomplete data, we will contact the author of the communication to obtain the missing data. If it is never available, exclude the experiment from the analysis.

#### 2.5.7 Assessment of heterogeneity

We will use the ReviewManager to assess efficacy and publication bias (version 5.3, Nordic Cochrane Centre, Copenhagen, Denmark). The forest map is used to illustrate the relative strength of the effect. The funnel plot is used to illustrate the bias because the number of trials exceeds 10. If a significant difference is detected, a random effects model will be used.

#### 2.5.8 Assessment of reporting biases

We will use a funnel plot to detect report bias. If more than 10 trials are included, the funnel plot will be used to assess the reported bias. If the funnel plot is found to be asymmetrical, analyze the cause using the Egger method. We will include all eligible trials regardless of the quality of the method.

#### 2.5.9 Data synthesis

We will use RevMan for all statistical analysis. If considerable heterogeneity is observed, a 95% CI random effects model will be used to analyze the combined effect estimates. Subgroup analysis will be performed with careful consideration of each subgroup if necessary.

#### 2.5.10 Subgroup analysis

There is no presubgroup plan. Subgroup analysis was performed based on control interventions and different outcomes.

#### 2.5.11 Sensitivity analysis

Based on sample size, heterogeneity quality, and statistical models (random or fixed-effect models), we will perform sensitivity analysis.

#### 2.5.12 Grading the quality of evidence

The quality of evidence for all outcomes will be judged by the grading of recommendations assessment, development, and evaluation working group approach. Bias risk, consistency, directness, precision, publication bias, and additional points are aspects of our assessment. High, medium, low, or very low represents the 4 levels of evaluation.^[50]^

## 3 Discussion

CU is an irritating and anxious condition of skin allergies, with more medical expenses and affecting work.^[10,14]^ Although it is benign, it can sometimes be a dangerous sign of serious medical illness. Therefore, this has caused more attention.^[51]^

Most studies suggest that mast cell degranulation is the main mechanism of its pathogenesis.^[17]^ The main method of treatment remains H1 antihistamine.^[3]^ Domestic and foreign research has proven that alternative treatments such as AWB or AS acupoint injection therapy are effective and safe for the treatment of CU, with operability, low cost, and broad prospects.^[32–35]^ Symptoms of CU should be considered for AWB or AS acupoint injection therapy, although further evidence of a placebo-controlled trial is needed.

The evaluation of this systematic review will be divided into 4 parts: identification, the inclusion of literature, data extraction and comprehensive analysis of data. According to the Cochrane method, this study is based on the analysis of clinical RCT evidence at home and abroad, searching and screening the main electronic literature database with evidence-based medical evidence, providing clinicians with more convincing evidence in decision-making, to better guide clinical treatment.

## Author contributions

**Conceptualization:** Leixiao Zhang, Ruting Hui, Yanli Deng, Ying Li.

**Methodology:** Qianhua Zheng, Siyuan Zhou, Ying Li.

**Software:** Hui Zheng, Pingsheng Hao, Ying Li.

**Supervision:** Wei Cao, Ying Liu, Ying Li.

**Validation:** Junpeng Yao, Ying Li.

**Writing – original draft:** Leixiao Zhang, Xianjun Xiao, Ruting Hui, Yunzhou Shi.
